# Supplementary material for: Direct visualization of stacking-selective self-intercalation in epitaxial Nb1+xSe2 films
Source: Nat Commun. 2024 Mar 21;15:2541. doi: 10.1038/s41467-024-46934-0 (PMC10957900; doi:10.1038/s41467-024-46934-0)
Supplement: Supplementary file 1 — Supplementary Information [file 41467_2024_46934_MOESM1_ESM.pdf]

Supplementary information

Direct visualization of stacking-selective self-intercalation in epitaxial $Nb_{1+x}Se_2$ films

Hongguang Wang[1,*], Jiawei Zhang[1], Chen Shen[2,*], Chao Yang[1], Kathrin Küster[1], Julia Deuschle[1], Ulrich Starke[1], Hongbin Zhang[2], Masahiko Isobe[1], Dennis Huang[1,*], Peter A. van Aken[1], Hidenori Takagi[1,3,4]

1. Max Planck Institute for Solid State Research, Heisenbergstr. 1, 70569, Stuttgart, Germany
2. Department of Materials and Earth Sciences, Technical University of Darmstadt, Darmstadt, Germany
3. Institute for Functional Matter and Quantum Technologies, University of Stuttgart, 70569 Stuttgart, Germany
4. Department of Physics, University of Tokyo, 113-0033 Tokyo, Japan

*Corresponding authors: D.Huang@fkf.mpg.de, chenshen@tmm.tu-darmstadt.de, hgwang@fkf.mpg.de

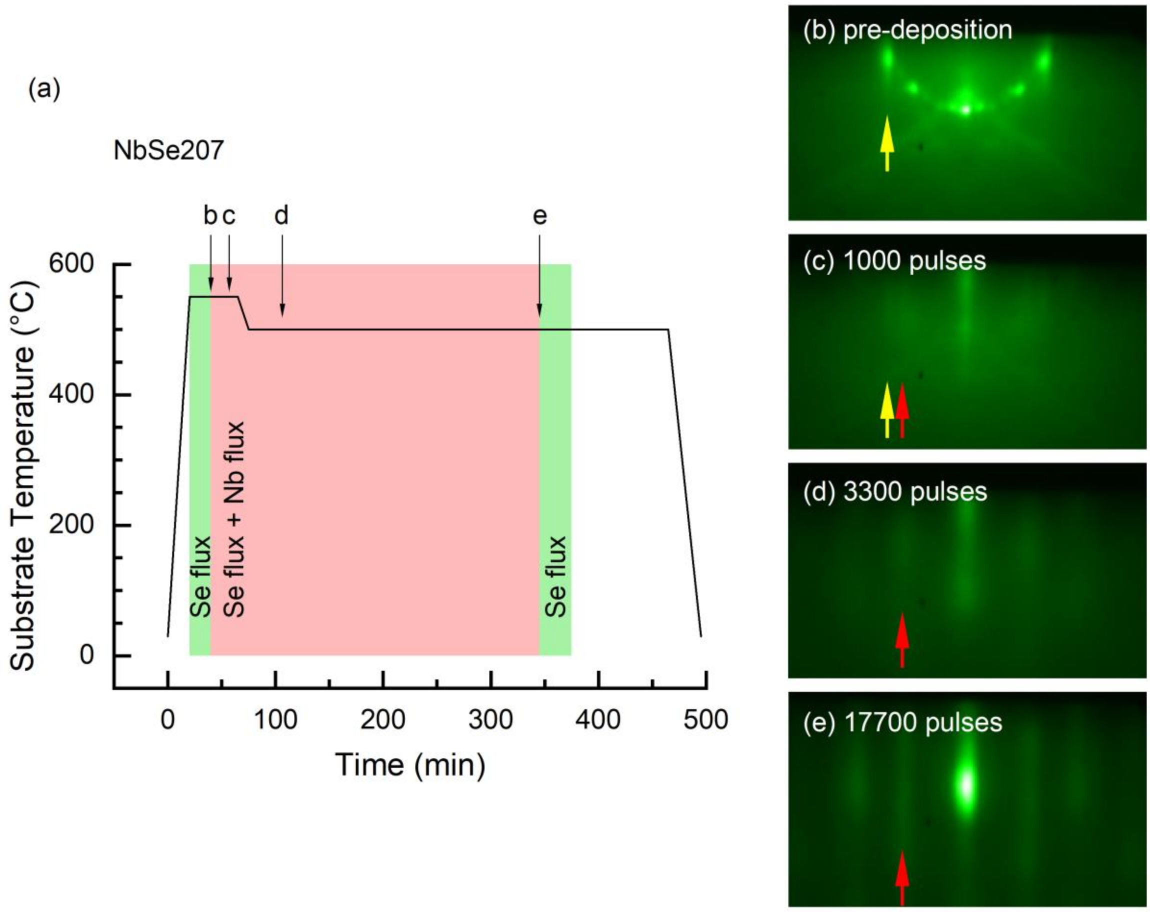

Supplementary Fig. 1 Growth process and *in-situ* RHEED monitoring (sample NbSe207). (a) The substrate temperature during growth versus time. The time slots when panels (b)–(e) were shot are marked with arrows. (b)–(e) RHEED patterns at different stages of the growth. Signals from the substrate are marked in (b), (c) with yellow arrows, while signals from the film are marked in (c)–(e) with red arrows. Electron energy: 10 keV. Direction: [1-100].

Supplementary Table 1 Growth parameters of $Nb_{1+x}Se_2$ films. [a]Deposition of the first layer; [b]deposition of the subsequent layers; [c]post-growth annealing. The spot size of the laser pulse on the target is estimated to be 1 mm^2.

Sample name	Substrate temperature [°C]	Laser fluence [J/cm^2]	Pulse count	Repetition rate [Hz]	Estimated thickness [layers]	Corresponding measurements
**NbSe163**	550^{abc}	7.2	72000	5	56	AFM
**NbSe164**	400^{a}, 600^{bc}	6.6	21600	1	14	AFM
**NbSe168**	550^{abc}	8.8	6000	1	5	AFM
**NbSe169**	550^{abc}	7.0	5000	1	2	AFM
**NbSe180**	550^{a}, 500^{bc}	8.5	17400	1	5–6	STM, XRD
**NbSe183**	550^{a}, 500^{bc}	6.0	16200	1	6–7	ARPES, LEED, XPS
**NbSe194**	550^{a}, 500^{bc}	4.6	15000	1	8–9	STEM, transport, STM, XRD
**NbSe207**	550^{a}, 500^{bc}	3.6	17700	1	7	STEM
**NbSe208**	450^{abc}	3.0	11800	1	5–6	ARPES, LEED, XPS

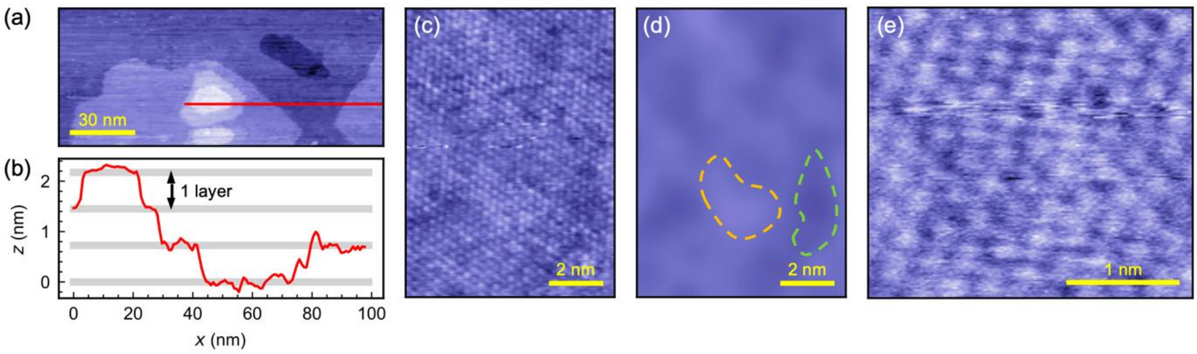

Supplementary Fig. 2 STM measurements. (a) STM topographic image of a $Nb_{1+x}Se_2$ film (sample NbSe180) acquired at room temperature. Set point: 1 V, 20 pA. (b) Row-averaged height profile across the red line in (a). (c) Atomically resolved topographic image (reproduced from Fig. 1(d) of the main text). Each bright spot corresponds to the topmost Se atom in a Se-Nb-Se layer. Set point: −125 mV, 50 pA. (d) Gaussian convolution of (c) with a width of 15 pixels, revealing the background inhomogeneity in (c). (e) Atomically resolved topographic image of a second film (sample NbSe194). Set point: 250 mV, 100 pA.

Supplementary Figs. 2(a) and 2(b) show a topographic image and a line cut across multiple step edges. Higher resolution images over smaller fields of view reveal a hexagonal atomic lattice of Se atoms [Supplementary Figs. 2(c) and 2(e)]. STM images such as these ones can reveal the presence of various defects, including vacancies, adatoms, and intercalants. Vacancies would appear as missing bright spots in the hexagonal lattice, whereas adatoms would appear as distinct, large bright spots on top of the hexagonal lattice. What we observe are not vacancies or adatoms, but an inhomogeneous background to the hexagonal lattice, with brighter and darker patches. To better visualize this background inhomogeneity, a Gaussian convolution was applied in Supplementary Fig. 2(d) to remove the atomic corrugations. This disorder likely arises from the subsurface Nb intercalants[1] and is consistent with STEM images showing varying intensities at Nb intercalant sites. For example, the area enclosed by the orange dashed line has a brighter intensity and may correspond to a region with more Nb intercalants, whereas the area enclosed by the green dashed line has a darker intensity and may correspond to a region with fewer Nb intercalants. We can also deduce that the intercalants do not exhibit in-plane ordering, and are inhomogeneous on the length scale of nanometers.

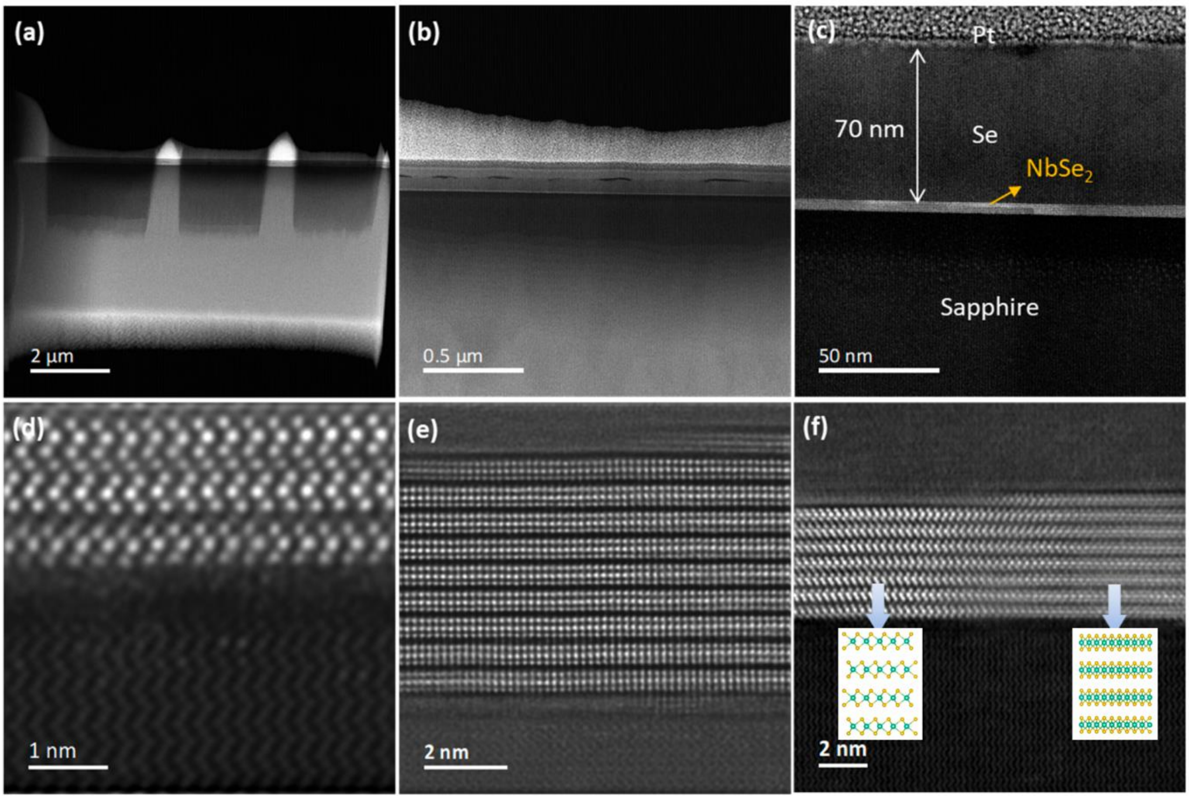

Supplementary Fig. 3 STEM studies. HAADF-STEM images of a $Nb_{1+x}Se_2$ thin film viewed along the [1-100] direction of the sapphire substrate. (a) Prepared FIB lamella of the $Nb_{1+x}Se_2$ thin film. (b) Low-magnification images of a thin region of the FIB lamella. (c) Overview image of the cross-section showing regions including the sapphire substrate, $NbSe_2$ layers, Se capping layer, and Pt protection layer. (d) Atomically resolved image at the interface between sapphire and $NbSe_2$. Here, we observe the [11-20] cross-section of the $NbSe_2$ layers. (e) Another region showing the [1-100] cross-section of the $NbSe_2$ layers. (f) Boundary region between $NbSe_2$ domains with different orientations.

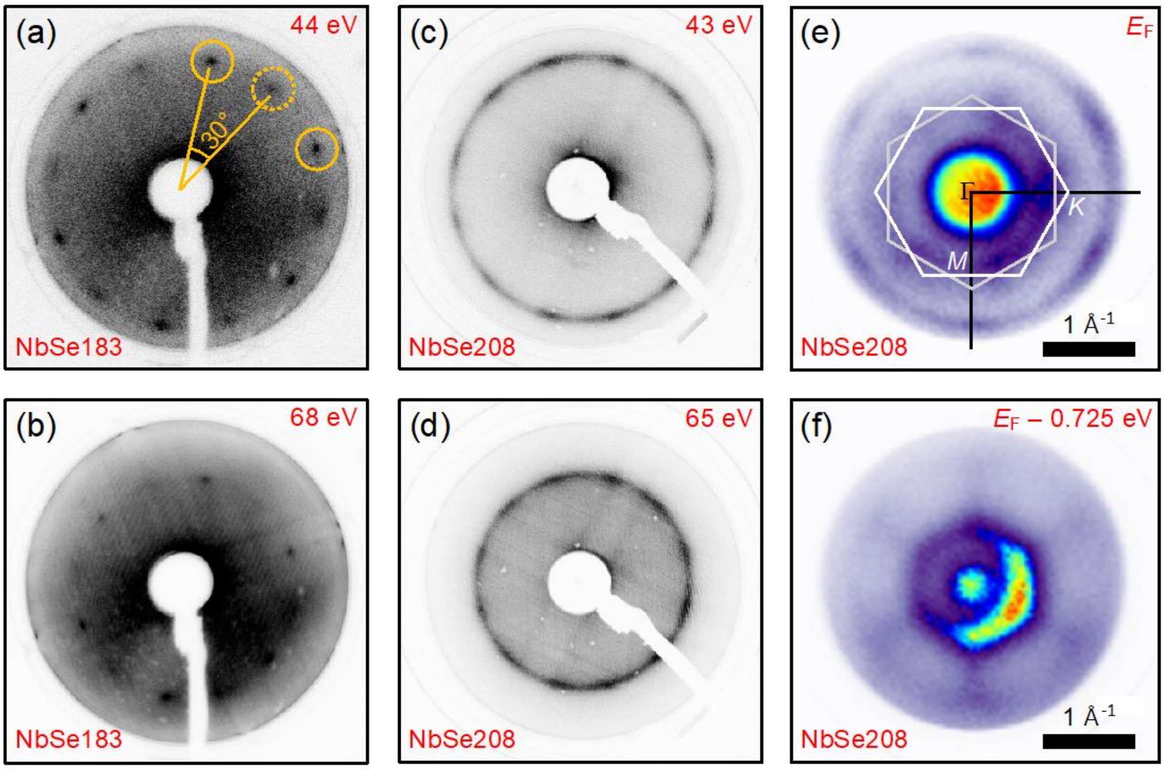

Supplementary Fig. 4 LEED results. LEED images of samples (a), (b) NbSe183 and (c), (d) NbSe208. The solid circles in (a) mark the dominant diffraction spots from the hexagonal $NbSe_2$ lattice, whereas the broken circle marks secondary diffraction spots from domains rotated by 30°. The electron energies are shown in the upper right corner of each panel. (e), (f) ARPES constant energy cuts of sample NbSe208 at the Fermi energy E_F and –0.725 eV below E_F. In (e), the Brillouin zone and its 30° rotations are overlaid.

Supplementary Fig.4 shows low-energy electron diffraction (LEED) images of two $Nb_{1+x}Se_2$ films, NbSe183 and NbSe208, acquired at two electron energies each. Since the films are 6–10 layers thick, we only observed diffraction spots from the hexagonal $NbSe_2$ lattice, and none from the Al_2O_3 substrate. As seen in Supplementary Fig. 4(a), there is a series of six primary diffraction spots (solid circles), as well as another series of secondary diffraction spots (broken circles), rotated by 30°. The secondary diffraction spots correspond to domains that are rotated by 30°, which were also seen in the STEM cross-sectional images (Figs. 1(e) and 1(f) of the main text, as well as Supplementary Fig. 3 and 5). Such domain rotation is common in layered vdW compounds grown via molecular beam epitaxy[1, 2]. For sample NbSe208 [Supplementary Figs. 4(c) and (d)], we could not distinguish the primary and secondary diffraction spots, and in addition, a ring-like background is apparent. The stronger rotational disorder may be due to the lower substrate temperature used during the growth of this film (450°C, compared to 550°C for NbSe183). This rotation disorder affects the ARPES cube data [Supplementary Figs. 4(e) and 4(f)], causing the band dispersions along Γ–*M*–Γ and Γ–*K*–*M* to be mixed.

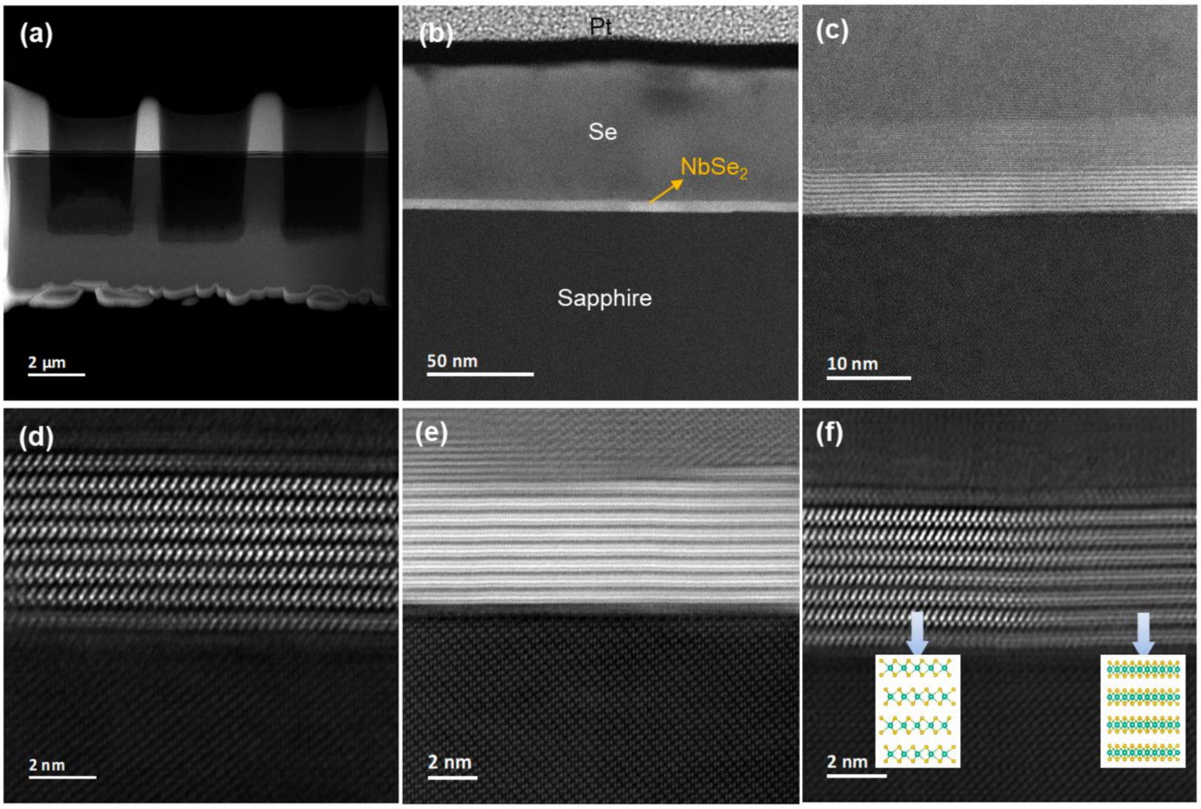

Supplementary Fig. 5 HAADF-STEM images of a $Nb_{1+x}Se_2$ thin film viewed along the [11-20] direction of the sapphire substrate. (a) Prepared FIB lamella of the $Nb_{1+x}Se_2$ thin film. (b) Overview image of the cross-section showing regions including the sapphire substrate, $NbSe_2$ layers, Se capping layer, and Pt protection layer. (c) Cross-section of overall NbSe layers. (d), (e) correspond to atomically resolved images of $NbSe_2$ layers in different orientations, along their [11-20] and [1-100] axes, respectively. (f) Boundary region between $NbSe_2$ domains with different orientations.

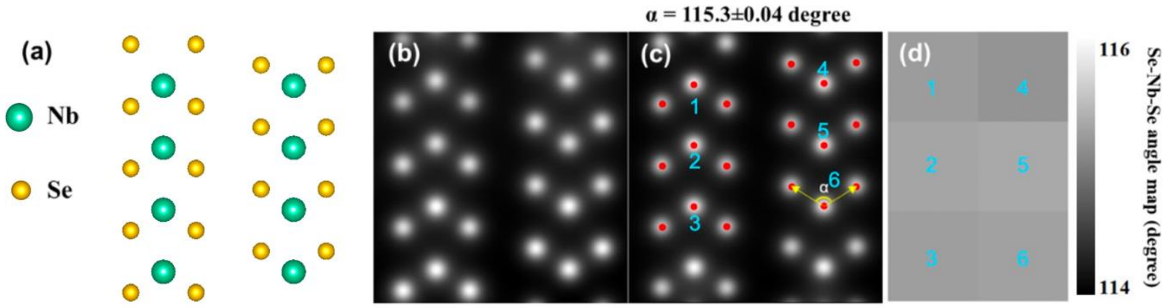

Supplementary Fig. 6 Quantification of the Se-Nb-Se angle. (a) Schematic for the pristine $2H_a$ phase of $NbSe_2$. (b) Simulated HAADF-STEM image with abTEM.[3] The structural file can be downloaded from the following link[4]: https://materials.springer.com/isp/crystallographic/docs/sd_0553177. (c) Fitted position for the atomic columns using a combination of 2D Gaussian fitting and center-of-mass methods. (d) The calculated 2D map of Se-Nb-Se angles.

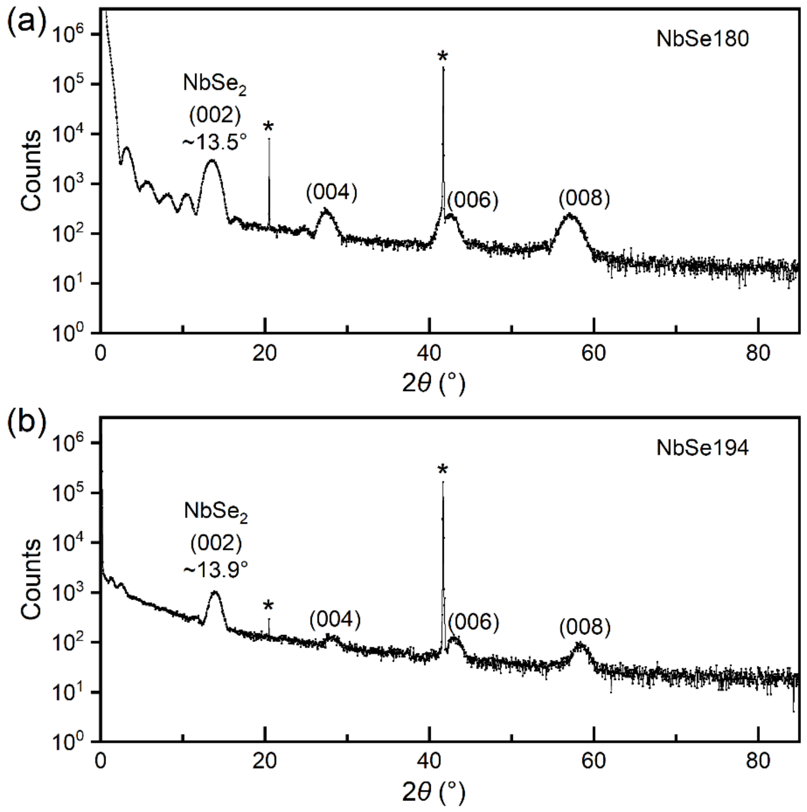

Supplementary Fig. 7. XRD θ-2θ scan of intercalated films (NbSe180 and NbSe194). Peaks from the sapphire substrate are marked as asterisks.

The XRD (x-ray diffraction) results of $Nb_{1+x}Se_2$ samples NbSe180 and NbSe194 are shown in Supplementary Fig. 7. Bragg reflections of the $NbSe_2$ layers are marked, and the contributions of the substrate are also observed. From the (002) reflection, we compute the average interlayer distances of samples NbSe180 and NbSe194 to be 6.55 and 6.37 Å, respectively. These values are larger than that of 6.27 Å in bulk $NbSe_2$ and are consistent with an expansion of the interlayer distance due to self-intercalation in $Nb_{1+x}Se_2$ [5]. Although the STEM images resolve a range of interlayer distances depending on whether the vdW gap is intercalated (180° stacking) or not (0° stacking) and how many intercalants there are, XRD, which averages over numerous domains within a macroscopic area, only resolves an overall increase in the average interlayer distance. Kiessig fringes are also observed at low 2θ-values. Kiessig fringes appear as oscillations in the XRD spectrum of thin films. The thicknesses of NbSe180 and NbSe194 can be roughly estimated as 5.5 layers and 10 layers, respectively.

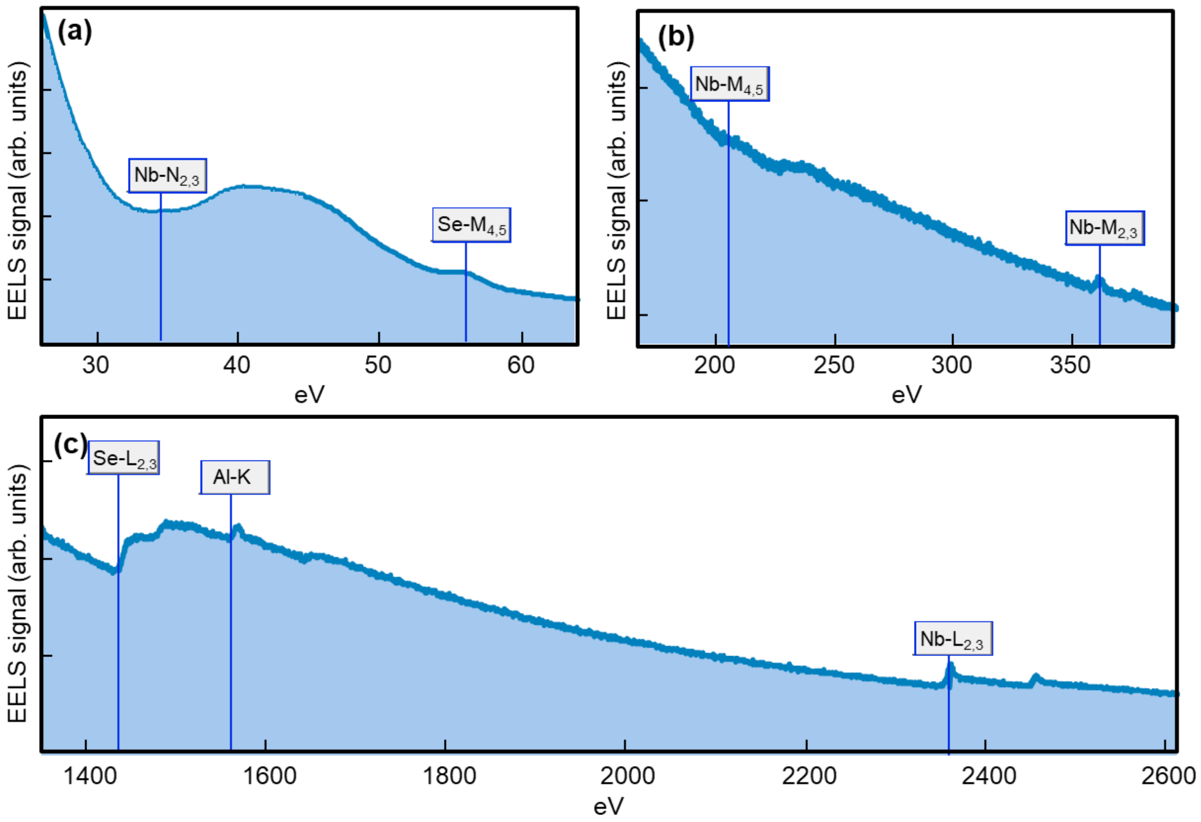

Supplementary Fig. 8 Collected EELS spectra. Characteristic features of the Nb-$N_{2,3}$ and Se-$M_{4,5}$ edges in (a), the Nb-$M_{4,5}$ and $M_{2,3}$ edges in (b), and the Se-$L_{2,3}$, Al-K, and Nb-$L_{2,3}$ edges in (c).

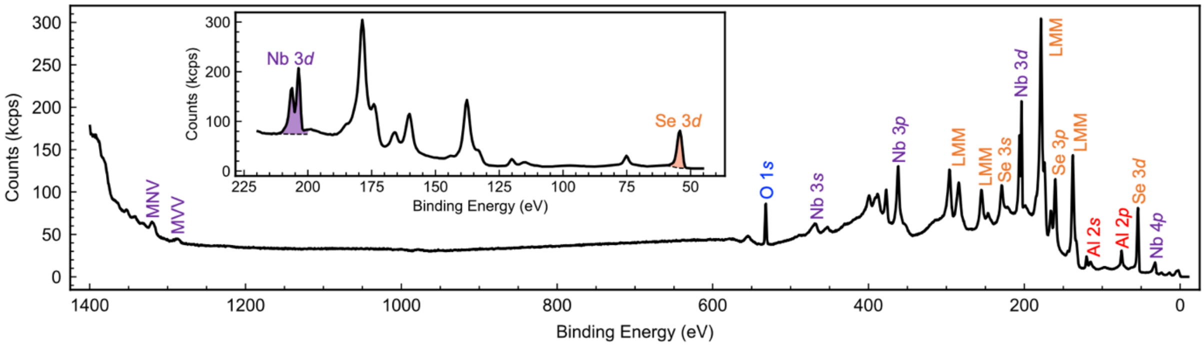

Supplementary Fig. 9 XPS survey spectrum of $Nb_{1+x}Se_2/Al_2O_3(0001)$, with photoelectron and Auger lines labeled (sample NbSe183). Inset: zoom-in around lower binding energies. The background used to estimate the areas underneath the Nb 3*d* and Se 3*d* core levels are indicated by the dashed lines.

Supplementary Fig. 9 presents the XPS survey spectrum of a $Nb_{1+x}Se_2$ film. By comparing the ratio of the areas underneath the Nb 3*d* and Se 3*d* core levels, we estimate a stoichiometry of $Nb_{1.29}Se_2$; i.e., x = 0.29, using the Scofield crosssections implemented in CasaXPS.

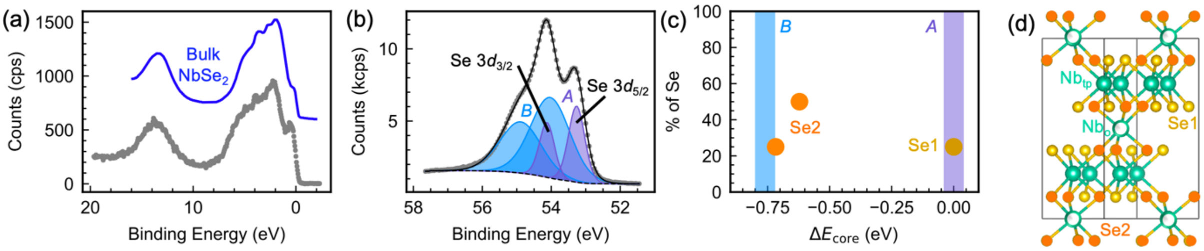

Supplementary Fig. 10 XPS analysis. (a) XPS valence band spectrum of a $Nb_{1+x}Se_2$ film (sample NbSe183). A corresponding spectrum for bulk $NbSe_2$ is shown for comparison (reproduced from Ref.[6]). (b) Core level spectrum of the Se $3d_{3/2}$ and $3d_{5/2}$ doublet. The gray circles are the measured data, the black line is the overall fit, the dotted line is the background, and the shaded areas are the individual peaks that constitute the fit. Photon source: monochromatized Al Kα. (c) Core-level shifts ΔE_{core} computed via DFT for a $Nb_{10}Se_{16}$ supercell, corresponding to an intercalant concentration of $x = 0.25$. The shifts of the *A* and *B* components relative to *A*, extracted from (b), are overlaid as vertical bars. (d) Structure of $Nb_{10}Se_{16}$ supercell with two coordinations of Se atoms (threefold, Se1, and fourfold, Se2) and two coordinations of Nb (trigonal prismatic, Nb_{tp}, and octahedral, Nb_o).

Supplementary Fig. 10 presents the XPS valence band and Se 3*d* core level spectra of a $Nb_{1+x}Se_2$ film. The overall shape of the valence band spectrum is qualitatively similar to that of bulk $NbSe_2$ [Supplementary Fig. 10(a)]. The Se $3d_{3/2}$ and $3d_{5/2}$ doublet is made of two components, labeled *A* and *B* in Supplementary Fig. 10(b). The two Se components, which are also seen in other intercalated TMDC compounds, arise, because the intercalant atom, Nb in this case, creates two crystallographic Se sites[7, 8]. In a pristine $NbSe_2$ compound, each Se is coordinated with three Nb atoms. When a Nb intercalant atom is inserted into the octahedral void of the vdW gap, it causes its six neighboring Se atoms to become coordinated with four Nb atoms, resulting in a core-level shift of those Se atoms. Component *A* corresponds to the threefold-coordinated Se atoms, whereas component *B* corresponds to the fourfold-coordinated Se atoms neighboring a Nb intercalant. From the area ratios of *A* and *B*, we can estimate the concentration of Nb intercalants, *x*, as follows: the area percentage occupied by components *A* and *B* are 28.2% and 72.8%, respectively (see Supplementary Table 2). Each Nb intercalant causes six neighboring Se atoms, or three Se atoms per $NbSe_2$ layer, to shift from component *A* to *B*. Then *x* is given by 0.728/3 $\approx$ 0.24, which is close to the first estimate by considering the area ratios of the Nb 3*d* and Se 3*d* core levels. As a caveat, this estimate is a lower bound, because it assumes that no pairs of Nb intercalants occupy nearest-neighbor sites of octahedral voids, which would result in fivefold- or even sixfold-coordinated Se atoms. In those cases, the number of intercalants would be larger than the number of fourfold-, fivefold-, and sixfold-coordinated Se atoms divided by three. So we estimate $x \geq 0.24$.

To confirm that component *B* originates from Se atoms neighboring a Nb intercalant with fourfold coordination, we used DFT to compute the core-level shifts of a $Nb_{10}Se_{16}$ supercell. The supercell has an intercalation concentration of precisely $x = 0.25$, with no nearest-neighbor pairs of Nb intercalants [see crystal structure in Supplementary Fig. 10(d)]. As seen in Supplementary Fig. 10(c), the energy difference between the core levels of threefold-coordinated Se atoms (Se1) and fourfold-coordinated Se

atoms (Se2) is around 0.6–0.7 eV, which is close to the energy difference of components *A* and *B*, 0.79 eV. Note that there are two values for Se2 in Supplementary Fig. 10(c), because there are two crystallographically inequivalent Se sites with fourfold coordination. Furthermore, the percentages of Se1 and Se2 atoms are 25% and 75%, respectively, which would yield $x = 0.75/3 = 0.25$.

	Se $3d_{5/2}$		Se $3d_{3/2}$	
	Se^{A}	Se^{B}	Se^{A}	Se^{B}
Line shape	GL(30)	GL(30)	GL(30)	GL(30)
Area [arb. Units]	3194.05	8144.83	2129.37	5429.89
Area [%]	14.30	37.45	13.88	35.36
Position [eV]	53.27	54.03	54.13	54.89
FWHM [eV]	0.56	1.35	0.52	1.41

Supplementary Table 2. XPS fitting parameters. GL(*p*) stands for a Gaussian-Lorentzian mixture function, where *p* is the mixing factor. $p = 100$ represents a pure Lorentzian, whereas $p = 0$ represents a pure Gaussian. FWHM stands for the full width at half maximum.

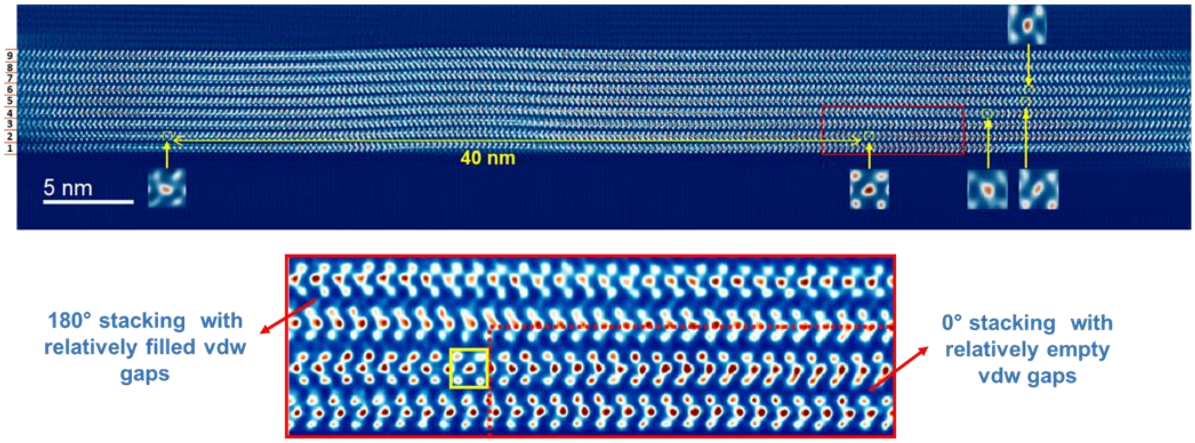

Supplementary Fig. 11 Atomic-resolution HAADF-STEM image of a region of the $Nb_{1+x}Se_2$ film (with a lateral length scale of around 60 nm). The yellow box marks the position where a lateral transition from 0° stacking to 180° stacking occurs. The close-up image of the red box region is shown in the bottom panel. The red dotted lines mark the boundary between the 180° and 0° stacking regions.

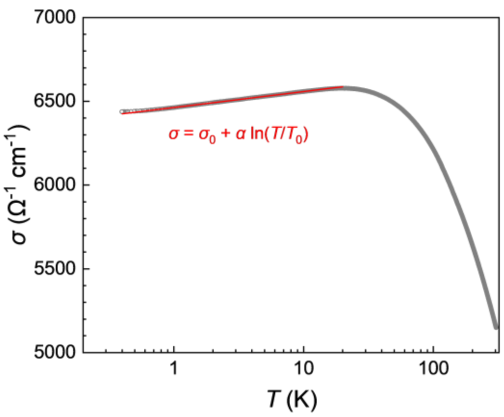

Supplementary Fig. 12 Conductivity of an intercalated thin film (NbSe194). The red line represents a linear fit with respect to logarthmic-scale temperature from 0.75 to 14 K.

Supplementary Fig. 12 shows the temperature dependence of the conductivity for the same sample presented in Fig. 4(f) of the main text. At low temperatures, the conductivity decreases exponentially, which can be described by the equation $\sigma = \sigma_0 + \alpha \ln(T/T_0)$. The fitting yields a value of $\alpha = 41.25\ \Omega^{-1}\ \mathrm{cm}^{-1}$. The contribution from one $NbSe_2$ layer is $\alpha^{2D} = \alpha * 0.625\ \mathrm{nm} = 2.58\times10^{-6}\ \Omega^{-1}$. For a 2D system, the decrease of conductivity contributed by the weak localization is expected to be[9]:

$$\delta\sigma = e^2/(\pi h)\ \ln(T/T_0),$$

Our measured value of $\alpha^{2D} \sim 0.21\ e^2/(\pi h)$ is close to this expectation.

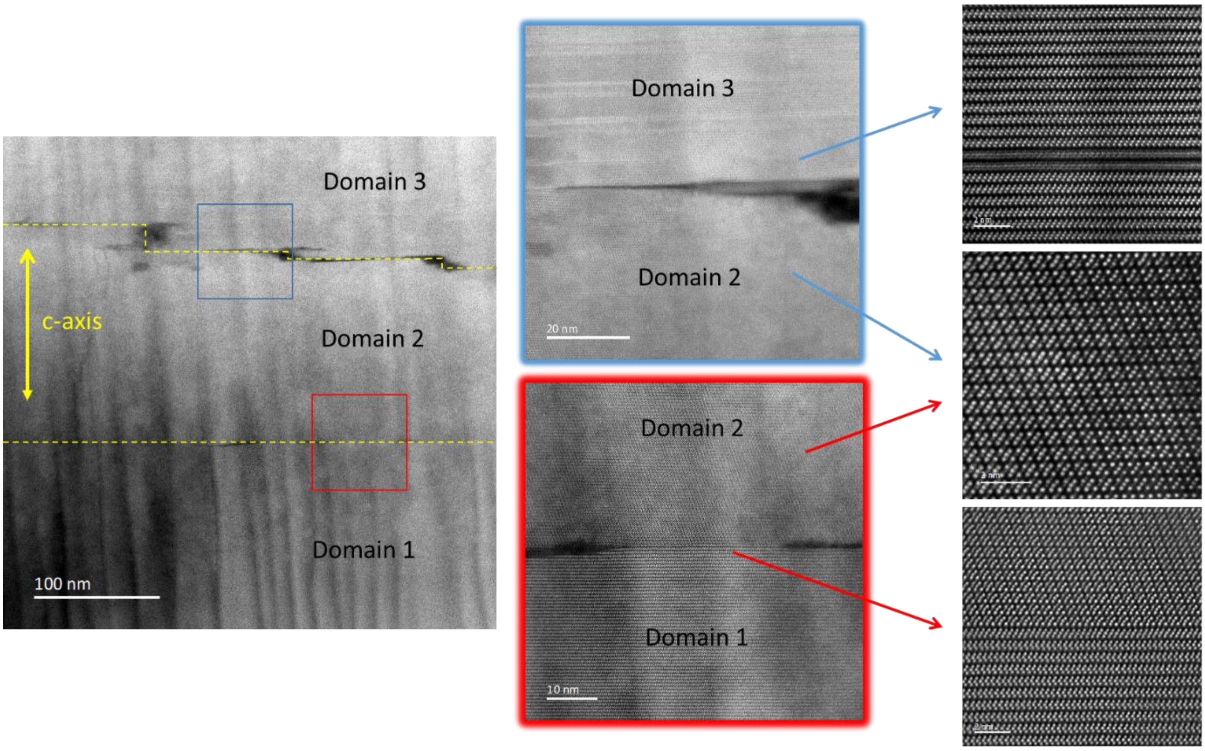

Supplementary Fig. 13 HAADF-STEM images of a cross-section of a $Nb_{1.20}Se_2$ crystal grown via CVT. In a field of view with dimensions of several hundreds of nanometers (left panel), we observed three domains. Domain 1 corresponds to layered 1*H*-$NbSe_2$ (trigonal prismatic coordination of Nb) stacked with 0° rotation, while domain 3 likely corresponds to layered 1*T*-$NbSe_2$ (octahedral coordination of Nb). Domain 2 contains an unidentified phase of Nb-Se.

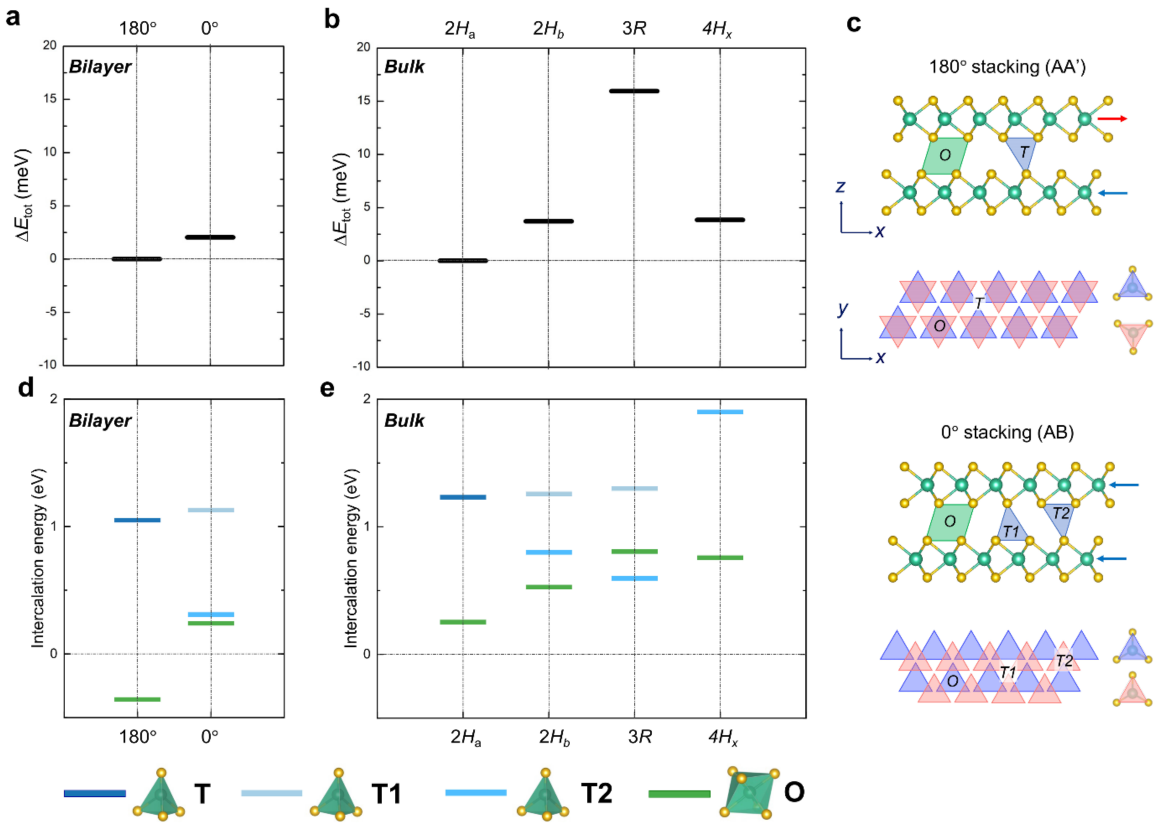

Supplementary Fig. 14 Additional DFT calculations of bilayer and bulk $Nb_{1+x}Se_2$. (a) The difference in DFT-computed total energy per atom, ΔE_{tot}, of pristine 0°-stacked bilayer $NbSe_2$ relative to 180°-stacked bilayer $NbSe_2$. (b) ΔE_{tot} of pristine $2H_b$, $3R$, and $4H_x$ bulk $NbSe_2$ relative to $2H_a$ bulk $NbSe_2$. (c) Octahedral (*O*) and tetrahedral (*T*) intercalation sites within the vdW gap of $NbSe_2$. For 0°-stacked layers, there are two inequivalent tetrahedral intercalation sites, *T1* and *T2*. (d) and (e) DFT-calculated change in total energy upon intercalation into various interstitial sites of the vdW gap of bilayer and bulk $NbSe_2$ with various stackings. The energies are computed for full occupancy (100%) of the intercalation sites, corresponding to Nb_3Se_4 for a bilayer slab and Nb_4Se_4 for a bulk unit cell. The blue and green bars denote intercalation energies at the tetrahedral and octahedral voids of the vdW gap, respectively. For the $4H_x$ polytypes, we could not stabilize an intercalant at the tetrahedral *T1* site.

Our DFT calculations in the main text [Figs. 6(a) and 6(b)] are focused on Nb intercalation of bilayer structures at the octahedral voids. Supplementary Fig. 14 presents additional DFT calculations including bulk structures and alternative intercalation sites. Supplementary Figs. 14(a) and 14(b) compare the relative energies of different stacking configurations without intercalation, and confirm that the $2H_a$ polytype has the lowest energy for both bilayer and bulk $NbSe_2$, though only by several meV. Supplementary Figs. 14(c)–(e) compare the intercalation energy, defined in the main text as $\Delta E_{int} = [E(Nb_{1+x}Se_2) - E(NbSe_2) - xE(Nb)]/x$, for various additional interstitial sites. At 100% intercalation, the octahedral voids within the $2H_a$ polytype are consistently the energetically favorable sites. We find that the energies for these 100%-intercalated bulk structures are correlated with their *c* axes, but anticorrelated with their *a* axes (Supplementary Fig. 15). It costs energy to expand the interlayer distance, and the Poisson ratio induces a simultaneous shrinking of the in-plane lattice constant. The more the intercalation causes the bulk structure to expand, the more energetically unfavorable it is.

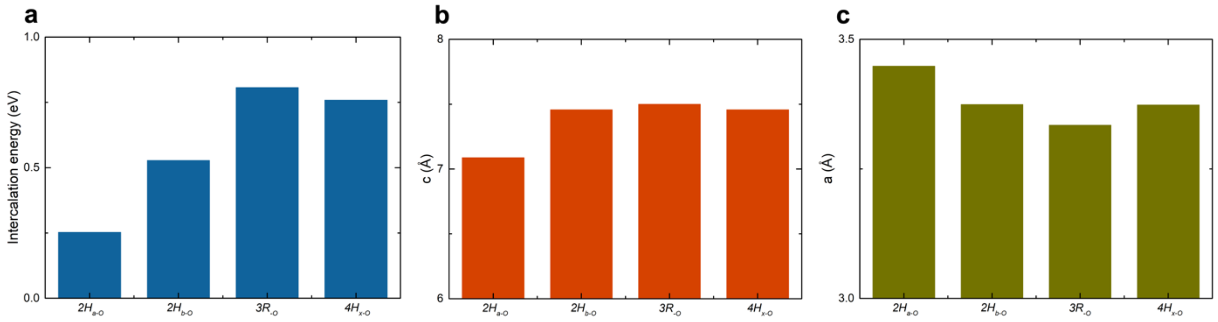

Supplementary Fig. 15 DFT results. Plots of (a) intercalation energy, (b) interlayer distance, (c) and in-plane lattice constant for various polytypes of bulk $NbSe_2$ with 100% intercalation at the octahedral site. The interlayer distance is correlated with the intercalation energy, whereas the in-plane lattice constant is anti-correlated with the interlayer distance.

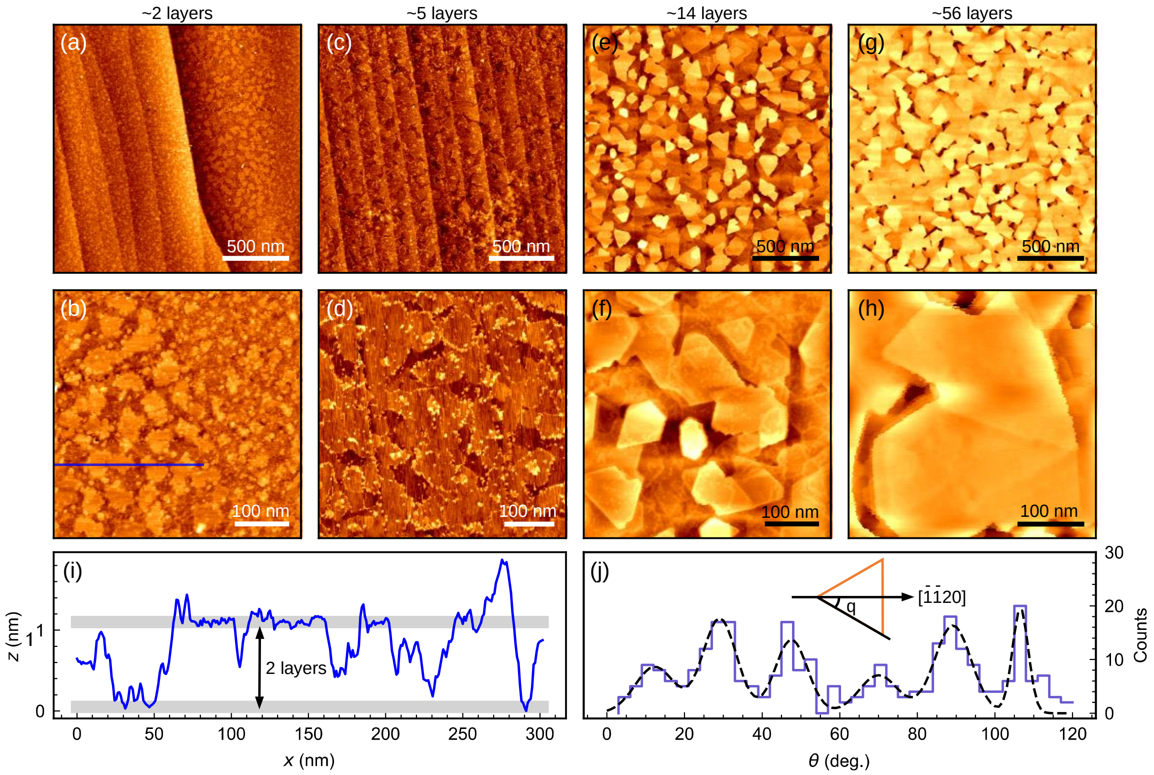

Supplementary Fig. 16 AFM results. AFM topographic images of $Nb_{1+x}Se_2$ films with increasing thickness: (a) and (b) ~2 Se-Nb-Se triple layers, (c) and (d) ~5 layers, (e) and (f) ~14 layers and (g) and (h) ~56 layers. (i) Height (z) profile across the blue line in (b). (j) Histogram of the rotational orientation of triangular islands in (e) and (g) combined. The dashed lines denote a fit to a sum of Gaussian functions. The inset defines the rotation angle θ.

We used atomic force microscopy (AFM) to characterize the morphology of $Nb_{1+x}Se_2$ films with an increasing number of layers. At a thickness of two [Supplementary Figs. 16(a) and 16(b)] and five [Supplementary Figs. 16(c) and 16(d)] Se-Nb-Se triple layers, the islands have round and irregular shapes. As shown in the line cut in Supplementary Fig. 16(i), the height of 2 layers is slightly less than $c = 1.254$ nm for bulk $NbSe_2$[10], likely because the distance between the first $NbSe_2$ layer and the sapphire substrate is less than the $NbSe_2$ interlayer spacing. At 14 and 56 layers [Supplementary Figs. 16(e)–16(h)], triangular islands have developed (sometimes with truncated edges), but strong in-plane mosaicity is observed. Supplementary Fig. 16(j) presents a histogram of island rotations derived from Supplementary Figs. 16(e) and 16(g), where θ is the in-plane angle relative to the direction perpendicular to the step edges, the [-1-120] axis of c-cut sapphire. Within the 120° range of rotation, we observed six dominant values of θ, whose values we extracted from a fit of the histogram to a sum of Gaussians: 12.3±1.6°, 29.1±0.7°, 47.5±0.8°, 70.0±1.7°, 89.2±0.7°, 106.5±0.4°. A rotation of 30° and 90° corresponds to the triangular islands having one edge aligned parallel to the edges of the terraces, which appears to be favorable. Such islands may have been nucleated from the step edges[11]. Interestingly, we did not observe many islands with θ = 0° (=120°) or 60°, as suggested by LEED measurements on

thinner films (<10 layers; Supplementary Fig. 4). Instead, we found islands with dominant rotations of ±17–19° away from 30° and 90°, suggesting that the domain rotation may evolve with film thickness.

In the AFM topographic images, the bright borders surrounding the islands [particularly visible in Supplementary Figs. 16(d) and 16(f)] are due to oxidation after exposure of the film to air. *In-situ* scanning tunneling microscopy (STM) measurements of films that were protected from air exposure revealed clean edges at the island, without such borders (Supplementary Fig. 2). In Supplementary Fig. 16(f), the bright lines extend within the triangular islands and likely delineate grain boundaries. These observations suggest that oxidation of $NbSe_2$ films begins at the exposed edges and grain boundaries.

Supplementary References

1. Lasek K, *et al.* Molecular Beam Epitaxy of Transition Metal (Ti-, V-, and Cr-) Tellurides: From Monolayer Ditellurides to Multilayer Self-Intercalation Compounds. *ACS nano* **14**, 8473-8484 (2020).

2. Ugeda MM, *et al.* Characterization of collective ground states in single-layer $NbSe_2$. *Nature Physics* **12**, 92-97 (2016).

3. Madsen J, Susi T. The abTEM code: transmission electron microscopy from first principles. *Open Research Europe* **1**, 24 (2021).

4. Kulikov LM, Kobzar' AAS, Yanaki AA, Akselrud LG, Koshel OS. Intercalation compounds of the composition $Zn_xNb_{1+y}Se_2$. *Inorganic Materials (translated from Neorganicheskie Materialy* **23**, 1729-1731 (1987).

5. Litwin PM, *et al.* The growth of self-intercalated $Nb_{1+x}Se_2$ by molecular beam epitaxy: The effect of processing conditions on the structure and electrical resistivity. *Journal of Vacuum Science & Technology A* **41**, (2023).

6. Wertheim GK, DiSalvo FJ, Buchanan DNE. Site inequivalence in $Fe1_{+x}Nb_{3-x}Se_{10}$. *Physical Review B* **28**, 3335-3338 (1983).

7. Huang D, *et al.* Probing the interlayer coupling in 2H-NbS_2 via soft x-ray angle-resolved photoemission spectroscopy. *Physical Review B* **105**, 245145 (2022).

8. Bonilla M, *et al.* Compositional Phase Change of Early Transition Metal Diselenide (VSe_2 and $TiSe_2$) Ultrathin Films by Postgrowth Annealing. *Advanced Materials Interfaces* **7**, 2000497 (2020).

9. Girvin SM, Yang K. *Modern condensed matter physics*. Cambridge University Press (2019).

10. Marezio M, Dernier PD, Menth A, Hull GW. The crystal structure of $NbSe_2$ at 15°K. *Journal of Solid State Chemistry* **4**, 425-429 (1972).

11. Chen L, Liu B, Ge M, Ma Y, Abbas AN, Zhou C. Step-Edge-Guided Nucleation and Growth of Aligned WSe_2 on Sapphire via a Layer-over-Layer Growth Mode. *ACS nano* **9**, 8368-8375 (2015).
